# Polymorphism of DNA Repair Genes via Homologous Recombination (HR) in Ovarian Cancer

**DOI:** 10.1007/s12253-019-00604-5

**Published:** 2019-02-02

**Authors:** Beata Smolarz, Magdalena M. Michalska, Dariusz Samulak, Hanna Romanowicz, Luiza Wójcik

**Affiliations:** 1 Laboratory of Cancer Genetics, Department of Pathology, Institute of Polish Mother’s Memorial Hospital, Rzgowska 281/289, 93-338 Lodz, Poland; 2 Department of Obstetrics and Gynaecology, Regional Hospital in Kalisz, Kalisz, Poland; 3 The State Higher Professional School of Stanisław Wojciechowski, Kalisz, Poland

**Keywords:** Ovarian cancer, DNA repair, Polymorphism, Gene

## Abstract

Ovarian cancer is one of the most common types of cancer in women. The repair system via homologous recombination repairs double-strand breaks (DSB) of DNA, which are the most mortal for cell, out of all DNA damages. The genes, which encode the double-strand break repairing proteins, are highly polymorphic and, taking into account the significance of the repaired defects for cancer development, it seems important to learn the role of the polymorphisms in ovarian cancer development. The aim of the study was to determine the relationship between DNA repair genes via homologous recombination (HR) and modulation of the risk of ovarian cancer. The following polymorphisms were analysed: *XRCC3*-Thr241Met (rs861539), *XRCC2*--41657C/T (rs718282), *XRCC2*-Arg188His (rs3218536), *BRCA1*-Q356R (rs1799950) and *RAD51*–135 G/C (rs1801320). The study group included 600 patients with ovarian cancer and 600 healthy controls. The PCR-RFLP (PCR-based restriction fragment length polymorphism) technique was applied for polymorphism analysis. Allele *XRCC3*-241Met (OR 0.85, 95%CI 0.72–0.99, *p* < 0.045), *XRCC2*-41657 T (OR 1.67, 95% CI 1.42–1.96, *p* < .0001), *BRCA1*-356R (OR 1.61; % CI 1.37–1.90, *p* < .0001) and *RAD51*–135C (OR 5.16; 95% CI 4.29–6.20, *p* < .0001) strongly correlated with the neoplastic disease. No relationship was observed between the studied polymorphisms and the cancer progression stage according to FIGO classification. The results indicate that polymorphisms of DNA repair genes via homologous recombination may be associated with the incidence of ovarian cancer. Further research on larger groups is warranted to determine the influence of above-mentioned genetic variants on ovarian cancer risk.

## Introduction

Ovarian cancer is the fourth most common cause of cancer among women in Poland. Because of the current lack of unequivocal molecular markers of ovarian carcinoma in its early stages, the primary objective of our study became an evaluation of the possibility to enrich the range of molecular markers, allowing for a more effective prognosis of ovarian cancer formation [1].

Cancer diseases are driven by a compromised DNA repair capacity [2]. The repair process usually encompasses two stages: the excision of lesion and the repair synthesis. This is how repair system act via base-excision repair (BER), nucleotide excision repair (NER), and mismatch repair (MMR) [3]. Totally converse is the repair system activity by direct lesion reversal, in which there is merely a single-stage process with maintained integrity of the DNA phosphodiester chain and the system of recombination repair. Defects of the proteins, which directly participate in DNA repair and its control, are associated with an increased susceptibility to malignant changes. The genes of the DNA lesion repair systems play the key role in maintaining the genome integrity and control the repair of mutation-affected DNA [4, 5]. Without the genes, DNA would continue the accumulation of errors which would within a short time prevent further cell survival. Proper DNA repair ensures genomic integrity and plays a significant role in its protection against effects of carcinogenic factors. The polymorphism of repair genes may influence the performance of the process, by which defects of genetic material are removed, thus influencing the individual susceptibility to formation of neoplastic disease [6].

The repair by recombination enables removal of a number of serious DNA lesions, including double-stranded breaks [7–9]. These breaks may bring about a loss of some chromosomes, causing translocation of genetic material between them. The repair pathway via homologous recombination allows for lesion removal, while ensuring high reproduction faithfulness of the primary sequence of modified DNA. A DNA molecule, characterised by sequential homology (usually, it is the undamaged homolog of the chromosome) is used as an array in the rep air process of damaged chromosome [10]. There were more than 130 DNA repair genes identified, in which a series of single nucleotide polymorphisms (SNPs) were discovered [11].

In order to define the role, which may be played by these variants in modulating the risk of cancer formation, it is necessary to define their functional significance. The variability, perceived in DNA repair genes, may be of clinical importance for evaluation of the risk of occurrence of a given type of cancer, its prophylactics and therapy.

We studied the relationship between the polymorphisms of DNA repair genes via homologous recombination and the predisposition to malignancies in the ovary. The following polymorphisms were analysed: *XRCC3*-Thr241Met (rs861539), *XRCC2*--41657C/T (rs718282), *XRCC2*-Arg188His (rs3218536), *BRCA1*-Q356R (rs1799950) and *RAD51*–135 G/C (rs1801320).

The goals of our studies included:

1. Searching for genetic polymorphisms which participate in DNA damage repair pathways via homologous recombination, which may lead to the risk of malignancy formation.
2. Comparison of DNA repair polymorphisms via homologous recombination in
  the process of neoplasia.
3. Evaluation of the significance of obtained study results as new risk factors for neoplasia in the Polish population.

## Materials and Methods

### Patients

Formalin-fixed paraffin-embedded (FFPE) tumor tissue specimens were obtained from ovarian cancer patients (*n* = 600), treated at the Departments of Gynaecology, Polish Mother’s Memorial Hospital - Research Institute in Lodz during the years 2003–2017. All the diagnosed tumors were assessed by criteria of the International Federation of Gynaecology and Obstetrics (FIGO). The study groups of patients were ethnically uniform, including females of the Polish origin, inhabitants of the Lodz Region. In addition 600 normal ovarian tissue (control samples) was obtained from women undergoing laparoscopy for non-malignant conditions. See Table 1 for a complete specification of the patients. Histopathological studies were carried out at the Department of Clinical Pathomorphology. The Local Ethic Committee approved the study and each patient gave written informed consent. (No 33/2015).

**Table 1:** The characteristics summary of ovarian cancer patients

Characteristics	Number of cases (%)	Number of controls (%)
Median age (range)	41.2 (37–79)	43.1 (35–77)
Menarche		
< 12 years old	270 (45.0%)	200 (30%)
> 12 years old	330 (55.0%)	400 (70%)
Number of pregnancy		
1	252 (42.0%)	240 (40%)
2–3	200 (33.3%)	180 (30%)
>4	148 (24.7%)	180 (30%)
Use of hormone replacement therapy - HRT		
Yes	410 (68.3%)	390 (75%)
No	190 (31.7%)	210 (35%)
Body mass index (BMI) (kg/m2)		
<24.9	200 (33.3%)	195 (32.5%)
25–29.9	240 (40.0%)	210 (35.0%)
>30	160 (26.7%)	195 (32.5%)
Family cancer		
Yes	310 (51.7%)	
No	290 (48.3%)	
Histology of tumour		
Serous	176 (29.3)	
Mucinous	48 (8.0)	
Endometrioid	149 (24.8)	
Clear cell	51 (8.5)	
Undifferentiated	131 (21.8)	
Other	45 (7.5)	
Grading		
G1	170 (28.3%)	
G2	405 (67.5%)	
G3	25 (4.2%)	
Size of tumor		
> 5 cm	170 (28.3%)	
< 5 cm	430 (71.7%)	
Tumour wall infiltration/injury		
Present	220 (36.7%)	
Absent	380 (63.3%)	

*n* = 600

### DNA Isolation

DNA was extracted using a QIAamp DNA FFPE Tissue Kit (Qiagen GmbH, Hilden, Germany) according to the manufacturer instruction.

### PCR-RFLP Analyses

Genomic DNA was isolated and the SNPs in DNA repair genes were determined by polymerase chain reaction-restriction fragment length polymorphism (PCR-RFLP) method. The reactive mixture, used when copying the fragments of all studied genes, had the same composition except the starting sequences (Table 2). Polymorphisms of the DNA repair gene was determined by PCR-RFLP, using the primers specified in Table 2. The 50 μL PCR mixture contained about 100 ng of DNA, 12.5 pmol of each primer, 0.2 mmol/L of dNTPs, 2 mmol/L of MgCl_2_ and 1 U of Taq DNA polymerase (TaKaRa, Japan). Amplification was carried out in a PTC-100 thermocycler of MJ Research Inc. (Walthman, MA, USA) in conditions, tailored to each of the selected genes (see Table 3).

**Table 2:** Primers used to analyze SNPs of the DNA repair gene

Gene	Polymorphism	Primer sequence	
		forward	reverse
*XRCC3*	Thr241Met	5′ACAGGGCTCTGGAAGGCACTGCTCAGCTCACGCACC3′	5′GCCTGGTGGTCATCGACTC3′
*XRCC2*	Arg188His	5’TGTAGTCACCCATCTCTCTGC3′	5′AGTTGCTGCCATGCCTTACA3′
*XRCC2*	-41657C/T	5′GGAGGCCGCAATGAGCTGAGATG3′	5′TCGGGAAGCTGAGGTGGGAGGA3′
*RAD51*	135G/C	5′TGGGAACTGCAACTCATCTGG3′	5′GCGCTCCTCTCTCCAGCAG3′
*BRCA1*	Q356R	5′-GGA CTC CCA GCA CAG AAAAA-3′	5′-TCC CCA TCA TGT GAG TCATC-3′

**Table 3:** Thermal cycler conditions for PCR and fragment lengths for polymorphic variants of the studied DNA repair genes

Gene	Polymorphism	Thermal cycler conditions	Restriction endonuclease	Fragments after digestion (bp)
*XRCC3*	Thr241Met	1. 95 ° C – 5 min 2. 95 ° C – 20 s 3. 63 ° C – 20 s 4. 72 ° C – 20 s 5. 2 → 3 → 4 × 35 cycles 6. 72 °C – 3 min	NcoI	Thr/Thr: 136 Thr/Met: 136, 97, 39 Met/Met: 97, 39
*XRCC2*	Arg188His	1. 95 ° C – 5 min 2. 95 °C – 20 s 3. 60 °C – 20 s 4. 72 °C – 20 s 5. 2 → 3 → 4 × 35 cycles 6. 72 °C – 3 min	HpnI	Arg/Arg: 290 Arg/His: 290, 148, 142 His/His: 148, 142
*XRCC2*	-41657C/T	1. 95 ° C – 5 min 2. 95 °C – 40 s 3. 72 °C – 45 s 4. 72 °C – 60 s 5. 2 → 3 → 4 × 35 cycles 6. 72 °C – 3 min	MvaI	C/C: 315, 59, 42, C/T: 357, 315, 59, 42 T/T: 357, 59
*RAD51*	135 G/C	1. 95 °C – 5 min 2. 95 °C – 20 s 3. 68 °C – 20 s 4. 72 °C – 20 s 5. 2 → 3 → 4 × 35 cycles 6. 72 °C – 3 min	MvaI	G/G: 86, 71 G/C: 157, 86, 71 C/C: 157
*BRCA1*	Q356R	1. 95 ° C – 3 min 2. 95 °C – 30 s 3. 57 °C – 45 s 4. 72 °C – 1 min 5. 2 → 3 → 4 × 35 cycles 6. 72 °C – 7 min	AluNI	R/R: 211 Q/Q: 134, 77 Q/R: 211, 134, 77

In order to obtain allelic variants of particular genes, the obtained PCR products were submitted to incubation with appropriate restrictive enzymes, revealing target sequences at polymorphic site within one of the variants. The PCR products, contained in 10 μl of the reactive mixture, were incubated for 16 h with 1 U of an appropriate restrictive enzyme (see Table 3). All the applied enzymes were products of the MBI Fermentas Company (Vilnius, Lithuania). Table 3 presents fragment lengths for various polymorphic variants of the studied DNA repair genes.

### Statistical Analysis

The effect of polymorphisms on the risk of cancer formation was evaluated by odds ratio (OR), together with 95% confidence interval (CI), obtained by means of a logistic regression model. The wild type of genotype and allele acted as reference.

In order to verify whether the Hardy-Weinberg principle is reflected in the studied populations, a specific computer program was applied, being available at the www.ihg.gfs.de (Institute of Human Genetics, Technical University Munich and Helmholtz Center Munich).

All the statistical tests were carried at the level of significance α = 0.05. In order to verify the hypothesis about the significance of age, body mass index (BMI), menarche, hormonal replacement therapy, and family cancer in the studied group, the χ2 analysis was used. *P*-values <0.05 were considered significant.

## Results

This study demonstrated that *XRCC3*-Met/Met genotype of Thr241Met polymorphism was statistically significantly correlated with ovarian cancer (Table 4). The Met/Met homozygote decreased the risk of cancer (OR 0.70; 95% CI 0.50–0.97, *p* = 0.041).

**Table 4:** Distribution of DNA repair genes genotype in patients and control group

	Patients (*n* = 600)		Controls (*n* = 600)			
*XRCC3-*Thr241Met	Number	(%)	Number	(%)	OR (95% CI)^a^	*p* ^*b*^
Thr/Thr	147	24.5	117	19.5	1.00 Ref	
Thr/Met	307	51.2	317	52.8	0.77 (0.57–1.03)	0.090
Met/Met	146	24.3	166	27.7	0.70 (0.50–0.97)	0.041
Thr	601	50.1	551	45.9	1.00 Ref	
Met	599	49.9	649	54.1	0.85 (0.72–0.99)	0.045
*XRCC2- -*41657C/T	Number	(%)	Number	(%)	OR (95% CI)^a^	*p*
C/C	150	25.0	180	30.0	1.00 Ref	
C/T	150	25.0	240	40.0	0.75 (0.56–1.01)	0.067
T/T	300	50.0	180	30.0	2.00 (1.50–2.66)	<.0001
C	450	37.5	600	50.0	1.00 Ref	
T	750	62.5	600	50.0	1.67 (1.42–1.96)	<.0001
*XRCC2-*Arg188His	Number	(%)	Number	(%)	OR (95% CI)^a^	*p*
Arg/Arg	177	29.5	190	31.7	1.00 Ref	
Arg/His	243	40.5	220	36.6	1.19 (0.90–1.56)	0.250
His/His	180	30.0	190	31.7	1.02 (0.76–1.36)	1.000
Arg	597	49.8	600	50.0	1.00 Ref	
His	603	50.2	600	50.0	1.01 (0.86–1.18)	0.920
*BRCA1-* Q356R	Number	(%)	Number	(%)	OR (95% CI)^a^	*p*
Q/Q	155	25.8	190	31.7	1.00 Ref	
Q/R	155	25.8	226	37.7	0.84 (0.62–1.13)	0.279
R/R	290	48.4	184	30.6	1.93 (1.46–2.56)	<.0001
Q	465	38.8	606	50.5	1.00 Ref	
R	735	61.2	594	49.5	1.61 (1.37–1.90)	<.0001
*RAD51–*135G/C	Number	(%)	Number	(%)	OR (95% CI)	*p*
G/G	89	14.8	147	24.5	1.00 Ref	
G/C	50	8.4	363	60.5	0.23 (0.15–0.34)	<.0001
C/C	461	76.8	90	15.0	8.46 (5.98–11.96)	<.0001
G	228	19.0	657	54.8	1.00 Ref	
C	972	81.0	543	45.2	5.16 (4.29–6.20)	<.0001

^a^Crude odds ratio (OR), 95% CI = confidence interval at 95%, ^b^Chi square

Arg188His and -41657C/T polymorphisms of *XRCC2* gene, respectively were analysed in patients. The prevalence of -41,657 T allele in those studies was statistically significantly higher in the group of patients with ovarian cancer vs. the control group (OR 1.67; 95% CI 1.42–1.96, *p* < .0001). The T/T homozygote may increase the risk of cancer (OR 2.00; 95% CI 1.50–2.66, *p* < .0001). The −41,657 T allele increased the risk of cancer progression (Table 4).

No statistically significant differences were observed in genotype frequencies of XRCC2 Arg188His polymorphism between the control group and the ovarian cancer patients (see Table 4).

A correlation was found of Q356R *BRCA1* gene polymorphism with ovarian cancer in the examined patients. Based on the obtained results, it was demonstrated that 356R allele predisposed to cancer development (OR 1.61; 95% CI 1.37–1.90, *p* < 0.001). The R/R genotype may increase the risk of cancer (OR 1.93; 95% CI 1.46–2.56, *p* < .0001).

The studies successfully demonstrated that 135C allele of *RAD51* gene 135G/C polymorphism was correlated with an increased risk of ovarian cancer in the studied population (OR 5.16; 95% CI 4.29–6.20, p < 0.001). The C/C homozygote may increase the risk of progression of the studied cancer (OR 8.46; 95% CI (5.98–11.96, *p* < 0.001).

No relationship was observed between the studied polymorphisms and the cancer progression stage acc. to FIGO classification.

The studied polymorphisms of DNA repair genes via HR were not associated with other risk factors, such as the body mass index (BMI), menarche, number of pregnancy and hormonal replacement therapy (*p* > 0.05).

## Discussion

The identification of cancer risk markers is one of major challenges for contemporary medicine. The observed imperfections of cancer prophylactics result, among others, from:

- the identification of pathology at the stage of morphological, and not molecular changes,
- possible technical and diagnostic errors,
- the necessity of frequent repetitions of diagnostic procedures,
- impossibility of pathology progression prognosis [12, 13]

At the actual level of medical knowledge, we are capable of dealing with almost any type of cancer, provided it is identified early enough. There are many factors which play a significant role in triggering cancer formation process, with genetic factors being of major significance. Ovarian cancer is characterised by the occurrence of different genetic changes in various genes [14–17]. Consequently, it is often not possible to give a straightforward answer to the question, whether these changes are more like causes or more like effects of the disease. If they are perceive as causes, it is justified to study if the genetic variability, observed in many populations and defined as genetic polymorphism, may in any way contribute to induction and/or development of malignant changes, including ovarian cancer. The discovery of genetic background for the cancer formation process is an extremely difficult challenge, as in the majority of cases, the reason lies in a combined activity of several factors.

A set of alleles of repair protein encoding genes may largely define the individual abilities for DNA damage repair, as well as the susceptibility to tumour development [18, 19]. The single nucleotide polymorphisms (SNPs) may change the risk of cancer disease. SNPs may then be regarded as potential markers of carcinogenesis [20, 21].

The repair system via homologous recombination repairs double-strand breaks (DSB) of DNA, which are the most mortal for cell, out of all DNA damages [7, 9].

Non-repaired DSBs cause a loss of chromosome fragments and, in consequence, cellular death. Accumulated DSBs lead to genome destabilisation and to its rearrangement. Disorders in genome DNA are accumulated with the advancing age of organisms, causing deregulation of transcription process, leading to cancer formation. The genes, which encode the double-strand break repairing proteins, are highly polymorphic and, taking into account the significance of the repaired defects for cancer development, it seems important to learn the role of the polymorphisms in ovarian cancer development.

As literature data demonstrate DNA damages to be highly significant in the pathogenesis of ovarian cancer, especially those which require repair by homologous recombination. Therefore, my studies were continued in an analysis of subsequent polymorphisms in the gene, which encoded the protein, participating in repair by homologous recombination.

The first stage of the study concentrated on an evaluation of *XRCC2* gene Arg188His and *XRCC3* gene Thr241Met polymorphisms. *XRCC2* and *XRCC3* genes belong to the DNA repair system via a homological recombination, which removes a number of serious DNA defects, such as, for example, two-strand breaks [22, 23].

A consequence of faulty repair may be a loss of some chromosomes and translocation of genetic material, what may lead to the development of cancers [8, 24–26].

Moreover, they are strong inducers of programmed cell death. The system of repair via homologous recombination allows for defect removal, ensuring high reproduction accuracy of the primary sequence of modified DNA. The cells, which are defective with regard to *XRCC2* and *XRCC3* genes, demonstrate a particular sensitivity to ionising radiation, UV and to factors which induce cross-bonding formation [27].

*XRCC2* Arg188His and *XRCC3* Thr241Met polymorphisms were selected for their documented participation in the pathogenesis of cancers. Following the data from world literature, both polymorphisms may increase the risk of occurrence of various neoplasms, including pancreas, ovary, breast, head and neck carcinoma [28–35].

The presented study demonstrated a significant association of studied Thr241Met polymorphism of *XRCC3* gene with the occurrence of ovarian cancer. In the reported study, the second investigated polymorphism, Arg188His of *XRCC2* gene, was not associated with ovarian carcinoma occurrence.

A subsequent stage of the study focused on 41657C/T polymorphism of *XRCC2* gene. It has been demonstrated in a number of reports that it plays a significant role in the development of neoplastic diseases, including ovarian cancer [36–39].

The presented studies, carried out within the population of Polish women, confirmed the relationship of the studied *XRCC2* gene polymorphism to the development of ovarian cancer.

The search for markers of ovarian cancer development was carried on in the further part of the study by an analysis of the polymorphisms of HR genes (*RAD51* and *BRCA1*). It was demonstrated in the literature reports that the 135G/C and Q356R polymorphisms of *RAD51* and *BRCA1* genes, respectively, were associated with an increased risk of cancer and influenced the histological malignancy grading [40, 41].

Earlier reports of many researchers, dealing with SNPs in *RAD51* gene, with my co-authorship as well, concentrated mainly on G135C and G172 T polymorphisms at 5′ region, not subject of translation. Since RAD51 participates in DNA repair, while also interacting with BRCA proteins, the mutations of which are often identified in ovarian cancer, the above-mentioned polymorphisms may be associated with a higher risk of this cancer development [42, 43].

Previous studies suggest that *RAD51* gene 135G/C polymorphism was associated with susceptibility to breast cancer and head-and-neck cancer [44, 45].

Recently, a variety of studies have focused on the association between the 135G/C polymorphism in the *RAD51* gene and ovarian cancer. However, the observed associations of these studies were inconclusive [46–48].

135G/C polymorphism can modify the way of mRNA splicing, what, in turn, affects the protein functions or the effectiveness of translation [49].

In spite of the abundance of results, there is still no unequivocal explanation of the role of RAD51 in cancer formation. Our assumption was such that another genetic variability could act either additively or independently of the above-mentioned polymorphisms in 5’UTR region, what may help explain the role of *RAD51* in ovarian cancer development. The presented study demonstrated a significant association of studied 135G/C and Q356R polymorphisms of *RAD51* and *BRCA1* genes, respectively, with the occurrence of ovarian cancer.

## Conclusion

A significant correlation was revealed between single nucleotide polymorphisms of DNA double-strand break repair genes via homologous recombination (HR) *XRCC3*-Thr241Met (rs861539), *XRCC2*--41657C/T (rs718282), *BRCA1*-Q356R (rs1799950) and *RAD51*–135 G/C (rs1801320) and ovarian cancer development.

Our research are affected certain limitations. The sample for the present study comprised of 600 patients. This sample is only a very small proportion of the entire population of ovarian carcinoma women in the country. Therefore the obtained results can not be considered as definitive and require further, more extensive evaluations, performed on bigger groups of patients.

To conclude, the SNPs within the analysed genes of DNA repair systems comprise the new potentially important group of risk factors of ovarian cancer development in the Polish women. The SNPs analysis may be used in the near future as a convenient method of selecting patients with high risk of morbidity. However, considering the equivocal results of the studies presented in the literature, further research in this field on larger groups of patients is recommended.

## Data Availability

Data will not be shared, because it is part of a clinical database.
